# Dietary fatty acids improve perceived sleep quality, stress, and health in migraine: a secondary analysis of a randomized controlled trial

**DOI:** 10.3389/fpain.2023.1231054

**Published:** 2023-10-25

**Authors:** Keturah R. Faurot, Jinyoung Park, Vanessa Miller, Gilson Honvoh, Anthony Domeniciello, J. Douglas Mann, Susan A. Gaylord, Chanee E. Lynch, Olafur Palsson, Christopher E. Ramsden, Beth A. MacIntosh, Mark Horowitz, Daisy Zamora

**Affiliations:** ^1^ Department of Physical Medicine and Rehabilitation, University of North Carolina at Chapel Hill School of Medicine, Chapel Hill, NC, United States; ^2^ Lipid Peroxidation Unit, Laboratory of Clinical Investigation, National Institute on Aging, Baltimore, MD, United States; ^3^ Department of Neurology, UNC School of Medicine, Chapel Hill, NC, United States; ^4^ Department of Medicine, UNC School of Medicine, Chapel Hill, NC, United States; ^5^ Intramural Program of the National Institute on Alcohol Abuse and Alcoholism, NIH, Bethesda, MD, United States; ^6^ Metabolic and Nutrition Research Core, UNC Medical Center, Chapel Hill, NC, United States; ^7^ Department of Psychiatry, UNC School of Medicine, Chapel Hill, NC, United States

**Keywords:** migraine, dietary intervention, fatty acids, sleep quality, stress, perceived health, quality of life

## Abstract

**Background:**

Migraine is a prevalent disabling condition often associated with comorbid physical and psychological symptoms that contribute to impaired quality of life and disability. Studies suggest that increasing dietary omega-3 fatty acid is associated with headache reduction, but less is known about the effects on quality of life in migraine.

**Methods:**

After a 4-week run-in, 182 adults with 5–20 migraine days per month were randomized to one of the 3 arms for sixteen weeks. Dietary arms included: H3L6 (a high omega-3, low omega-6 diet), H3 (a high omega-3, an average omega-6 diet), or a control diet (average intakes of omega-3 and omega-6 fatty acids). Prespecified secondary endpoints included daily diary measures (stress perception, sleep quality, and perceived health), Patient-Reported Outcome Measurement Information System Version 1.0 ([PROMIS©) measures and the Migraine Disability Assessment (MIDAS). Analyses used linear mixed effects models to control for repeated measures.

**Results:**

The H3L6 diet was associated with significant improvements in stress perception [adjusted mean difference (aMD): −1.5 (95% confidence interval: −1.7 to −1.2)], sleep quality [aMD: 0.2 (95% CI:0.1–0.2)], and perceived health [aMD: 0.2 (0.2–0.3)] compared to the control. Similarly, the H3 diet was associated with significant improvements in stress perception [aMD: −0.8 (−1.1 to −0.5)], sleep quality [aMD: 0.2 (0.1, 0.3)], and perceived health [aMD: 0.3 (0.2, 0.3)] compared to the control. MIDAS scores improved substantially in the intervention groups compared with the control (H3L6 aMD: −11.8 [−25.1, 1.5] and H3 aMD: −10.7 [−24.0, 2.7]). Among the PROMIS-29 assessments, the biggest impact was on pain interference [H3L6 MD: −1.8 (−4.4, 0.7) and H3 aMD: −3.2 (−5.9, −0.5)] and pain intensity [H3L6 MD: −0.6 (−1.3, 0.1) and H3 aMD: −0.6 (−1.4, 0.1)].

**Discussion:**

The diary measures, with their increased power, supported our hypothesis that symptoms associated with migraine attacks could be responsive to specific dietary fatty acid manipulations. Changes in the PROMIS© measures reflected improvements in non-headache pain as well as physical and psychological function, largely in the expected directions. These findings suggest that increasing omega-3 with or without decreasing omega-6 in the diet may represent a reasonable adjunctive approach to reducing symptoms associated with migraine attacks.

**Trial Registration:** ClinicalTrials.gov NCT02012790.

## 1. Introduction

Migraine is a common, painful disorder, second only to low back pain as a disabling condition in the United States (US) (1). Comorbid physical and psychological symptoms—including stress, perceived health, insomnia, anxiety, and depression—are prevalent and reduce quality of life among patients with migraine (1, 2). Many strategies for treating migraine improve the frequency and/or duration of attacks but may be associated with side effects that have a negative overall impact on quality of life. Hence, it is critical to consider all symptoms related to quality of life when judging the therapeutic effectiveness of strategies for reducing migraine.

Targeting dietary intakes of omega-3 and omega-6 fatty acid to address migraine-related pain is supported in previous studies. Lowering dietary omega-6 polyunsaturated fatty acids (PUFA), especially linoleic acid (LA) in the diet, has been shown to result in lower levels of circulating omega-6 PUFA derived lipid mediators with pro-nociceptive properties (3–5). In addition, increasing dietary omega-3 eicosapentaenoic acid (EPA) and docosahexaenoic acid (DHA) increases circulating omega-3 derived lipid mediators, such as omega-3 monoepoxides (6, 7) and resolvins (8), with anti-nociceptive effects. Increasing omega-3 in the diet and reducing omega-6 over 12 weeks (compared with reducing omega-6 alone) resulted in reductions in headache frequency in chronic daily headache (9).

In a three-arm, 16-week dietary intervention for chronic and episodic migraine, compared with a diet with average intakes of omega-3 and omega-6, increasing omega-3 in the diet with or without decreasing omega-6 resulted in fewer and shorter headaches (10). Based on the migraine literature, we expected that the burden of comorbid non-headache pain, disability, and psychological symptoms among the study participants with migraine would be high. We hypothesized that the two active intervention diets would result in an improvement in comorbid symptoms. Furthermore, we expected that symptom improvements would be related to changes in plasma omega-3 and omega-6 levels.

## 2. Materials and methods

### 2.1. Trial protocol

This 3-arm parallel-group, 16-week randomized, controlled trial sought to evaluate the biochemical and clinical effects of dietary interventions that manipulated omega-3 and omega-6 PUFA in a sample of individuals with chronic migraine and frequent episodic migraine. The interventions included a control diet consistent with Dietary Guidelines for Americans as well as a diet that increased omega-3 PUFA intakes and a diet that both increased omega-3 PUFA intakes and decreased omega-6 PUFA intakes. The trial was registered with ClinicalTrials.gov (NCT02012790) prior to recruitment of participants. Please see the protocol, diet methods, and primary outcomes papers for additional details regarding the composition of the diets and specific study procedures (10–12). In brief, the control diet recommendations included low saturated fat, but levels of omega-6 PUFA and omega-3 PUFA consistent with average intakes in the United States (11). The H3 diet increased omega-3 PUFA intakes but did not decrease omega-6 PUFA intakes compared with the control diet. The H3L6 diet increased omega-3 PUFA intakes and sought to decrease omega-6 intakes compared to the control diet. All three dietary interventions included provision of key foods to alter omega-3 and omega-6 intake, diet education and diet adherence counseling delivered by trained research dietitians every 2–3 weeks at randomization and at intervention weeks 2, 4, 7, 10, 13, and 16.

Most of the participants were recruited from headache specialty clinics. At the baseline visit, after participants reviewed, discussed, and signed the informed consent documents, they reviewed eligibility criteria with the study physician. Eligible participants were adults (18 years of age and older) of any gender or ethnicity with documented migraine under the care of a physician who had at least 5 migraine days per month and no more than 20 migraine days per month. Participants were excluded if they were pregnant or breast-feeding or if they had changed their hormone medication intakes within the past 6 months. Other exclusions included the following: (1) serious psychiatric illness or substance abuse; (2) major medical illness; (3) recent head/neck trauma or surgery; (4) cognitive impairment; (5) food allergy in adulthood; (6) aversion to fish; (7) regular exposure to fish oil supplements; (8) recent or intended weight loss or prior bariatric surgery; (9) earlier participation in a dietary intervention or recent participation in any migraine intervention trial.

### 2.2. Masking, randomization, and diet intervention

After a 4–6-week run-in period, participants who met the criteria for participation (5–20 migraine days per month) were randomized 1:1:1 to one of the three diets by the dietitian using an uneditable computer interface with randomized blocks to ensure concealed allocation. The computer sequence was generated by a co-investigator who maintained the diary but was otherwise not involved in the trial. Initially, recruitment was limited to 5–14 migraine days per month (with up to 20 headache days), but was liberalized (5–20 migraine days per month) to meet recruitment targets, thereby including individuals with chronic migraine, as defined in ICHD-3 (13, 14) (headaches 15 days per month for at least 3 months with migraine features on at least 8 days per month). Only the dietitian and participant were aware of diet assignment. Participants were unaware of trial hypotheses related to PUFA intakes and were introduced to the diets as potentially equally efficacious for reduction of headaches. All other study personnel, including research assistants and analysts were blinded to intervention assignment.

Participants met with the research dietitian in person at the clinical research center 7 times during the active intervention, receiving enough food for 2 meals and 2 snacks per day for 2–3 weeks, extensive dietary counseling, and access to a website that detailed grocery and restaurant guides, recipes, and other dietary education materials. Diets were designed to be as alike as possible, containing the same proportions of fat, carbohydrates, and protein across the three, differing only in fatty acid content and protein sources (11). For example, the high omega-3 diets contained fatty fish and the control diet contained low fat fish and chicken breast. Diets aimed to provide enough calories to maintain a participant's current weight. Weight was recorded at each study visit.

Participants completed electronic headache diaries throughout the study, supplied blood samples and completed questionnaires. The study was approved by the Institutional Review Board of the University of North Carolina at Chapel Hill in accordance with the Declaration of the World Medical Association.

### 2.3. Safety procedures

The safety of participants in the trial was both actively and passively monitored. At each intervention visit, the dietitian inquired about potential adverse events related to the diets including gastrointestinal symptoms, allergy symptoms, and weight changes. Adverse events were also monitored through the comments section of the electronic headache diary. Adverse event reports were made biannually to the Data and Safety Monitoring Board at the University of North Carolina at Chapel Hill. The relationship of an adverse event to the intervention was judged by a neurologist blind to intervention assignment.

### 2.4. Measures

#### 2.4.1. Demographics and physical characteristics

At baseline, characteristics that may have affected either level of participation or benefit from the diet were collected. These include age, sex, Body Mass Index (BMI), blood pressure, education, race/ethnicity, income level, and relationship status. We also collected the baseline values of the Headache Impact Test (HIT-6) as well as measures of headache frequency and severity and preventive medication use. These include potentially psychoactive medications such as anti-depressants and anti-anxiety drugs as well as other medications such as gabapentinoids and muscle relaxers which can have an impact on sleep, fatigue, and physical function.

#### 2.4.2. Intervention credibility

Although participants were not made aware of the hypotheses associated with diet interventions, they were aware of the details of their assigned diet. In this situation, it is recommended that investigators assess expectations associated with the interventions. This study used a credibility measure in common use in integrative health studies (15, 16). The measure was adapted for use in headache populations and administered after participants received diet instruction, but before any food intakes. It measured (0–9 scale, 9 most credible) how likely participants would be to recommend the diet to others, how important it would be to make the treatment available, and how successful the treatment seemed for treating associated symptoms, e.g., tension, anxiety, or insomnia (17).

#### 2.4.3. Measures of psychological distress and quality of life

Key clinical outcomes have been reported in a previous publication (10). These include the Headache Impact Test (HIT-6) as well as headache frequency, duration, and severity as measured in a daily headache diary. Here we report the additional prespecified clinical and biochemical endpoints.

#### 2.4.4. Electronic headache diary: measures of daily stress, sleep quality, and perceived health

Participants completed an electronic daily diary for at least 4 weeks before randomization and during the 16 weeks of the intervention. The password-protected diary could be accessed from a computer or smartphone and, to reduce bias, was limited to entries for the current and previous day. To promote adherence, if participants did not complete a diary entry for the day before by 6 PM, they received a text message prompt.

In addition to headache frequency, severity, and duration, the diary included a question about the participant's stress level during the previous day on a 0–10 scale with 10 representing the greatest level of stress. Two 4-level Likert-style questions were also included: a rating of overall health and a rating of sleep quality, both with responses of poor (1), fair (2), good (3), and excellent (4). The diary was developed by our group and tested in prior headache studies (18).

#### 2.4.5. Patient reported outcomes measurement information system (PROMIS-29)

The National Institutes of Health (NIH) commissioned the PROMIS measures to enable flexible cross-study comparisons of health-related quality-of-life with a first wave of testing from 2005 to 2008. The PROMIS-29 covers physical (physical function, pain intensity, pain interference, fatigue), emotional (anxiety, depression, social functioning), and sleep disturbance. The PROMIS measures have been validated in multiple populations, including the general public and chronic disease populations (19–21). Analysis involves a translation to T scores that are normed to the general population, enabling ready interpretation. *T* scores of 50 correspond to the population mean and the standard deviation is set at 10 for all measures except the pain intensity score. The pain intensity score is consistent with the numeric rating scale for average pain over the past 7 days, measured on a 0–10 scale. Higher scores on the physical function and social functioning scores reflect improvements and lower scores on the pain intensity, pain interference, fatigue, sleep disturbance, anxiety, and depression measures represent improvements. Minimal clinically important differences (MCID) have been reported provisionally as 3.0–3.5 *T*-score points for depression (22), 2–3 *T*-score points for pain interference (in a sample of individuals with chronic pain or osteoarthritis) (22, 23), 2.3–3.4 *T*-score points for anxiety (osteoarthritic population) (22), and 1.9–2.2 *T*-score points for physical function (osteoarthritis population) (22).

The Center for Medicare and Medicaid Services assigns G-codes to levels of functional impairment for the purposes of billing for therapy services. A few of the PROMIS measures have been mapped to G-codes (24), including physical function, pain interference, and fatigue. For example, a physical function score of 50 corresponds to 1%–19% impairment for physical function and pain interference, but to 20%–39% impairment for fatigue.

#### 2.4.6. Migraine disability assessment (MIDAS)

The MIDAS assesses the number of days over a 3-month period that a migraine sufferer is either unable to or limited in their ability to participate in work or social activities (44, 45). The MIDAS score consists of 5 summed scores consisting of (1) the number of days of missed school or work, (2) the number of days of halved productivity at school or work, (3) the number of days of missed household work, (4) the number of days of halved productivity in the household and (5) the number of days missed for social activities (25). A MIDAS score of 11–20 indicates moderate disability and a score >20 indicates severe (26). The MIDAS has been found to be reliable (test-retest *r* = 0.8) and highly correlated with a paper headache diary and physician assessments (25). Clinically meaningful changes in the MIDAS have been defined as a 5-point decrease (27).

#### 2.4.7. Perceived change in symptoms and satisfaction with care

Participants evaluate how their headache symptoms and overall health has changed over the course of the intervention using a Likert scale with responses ranging from much worse (5) to much better (1). Similarly, they provide assessments of their satisfaction with their clinical care with responses ranging from very dissatisfied (1) to very satisfied (5). The questions were designed to measure the overall perceptions of benefit of the interventions.

#### 2.4.8. Whole-body pain scale

The Whole-Body Pain Scale was developed by co-author Olafur Palsson for this study to assess pain in addition to headache as experienced in the previous 7 days. The scale measures pain intensity as mild (1), moderate (2), and severe (3) across multiple body parts and asks about the total percentage of time a person experiences pain (0%–100% of the time). For the purposes of this analysis, we are examining the number of total body parts impacted by pain without considering the severity of the pain. A second analysis addresses the percentage of time people have pain.

#### 2.4.9. Measures of plasma fatty acids

In the H3 and the H3L6 diet groups, omega-3 fatty acids were expected to increase in plasma substantially. In addition, the H3L6 group was expected to decrease L6. These fatty acid measures were expected to be associated with clinical outcomes. Fatty acids were extracted from plasma and quantified by gas chromatography coupled to a flame ionization detector as previously described (28). Reported here are the model-predicted values at baseline and 16 weeks, the pre-post difference, and the between-group differences (controlling for multiple comparisons). We also present the association between changes in plasma fatty acids (EPA, DHA, arachidonic acid, and linoleic acid) and the diary-based outcomes (daily stress, perceived health, and sleep).

### 2.5. Data analysis

Sample size estimates were calculated for the primary biochemical endpoint, 17-hydroxy-DHA and the primary clinical endpoint, the HIT-6, as reported previously (10). No adjustments are made for multiple outcomes or multiple comparisons.

We addressed missing data using longitudinal mixed effects models and, where appropriate, multiple imputation retaining all randomized individuals in their original groups. Initial examinations indicated that we could assume data were missing at random and that missingness could be predicted. We used within-group chained equations with predicted mean matching and 30 imputed datasets, using Rubin's rules to combine them. Imputation models included demographic and clinical characteristics, headache variables, sleep quality, stress, overall health, medication use, expectation of benefit, and recruitment site. For the fatty acid measures, we used a simple imputation, consisting of the last value carried forward for all participants with at least one post-randomization measurement.

We used analysis of covariance (ANCOVA) for analyses with two time points and mixed effects models for more than two time points, with model choices based on the distribution of the outcome data. For example, the PROMIS measures, using *T*-scores, were analyzed using linear models. The MIDAS, with a right skew, was transformed to achieve normality. Because the transformed data did not change the interpretation, the untransformed medians are reported along with untransformed differences in means with 95% confidence intervals. Mixed effects models included a random intercept for individual participants, a time variable, indicator variables for group assignment, and time-by-group interactions. All models controlled for recruitment site and the baseline level of the variable examined as prescribed in the protocol. Associations between changes in sleep quality, perceived health, stress and plasma fatty acids are reported in exploratory mixed effects models.

A sensitivity analysis examined the effects of the interventions across episodic vs. chronic migraine and migraine with and without aura.

## 3. Results

### 3.1. Baseline characteristics of sample

**Figure 1:**
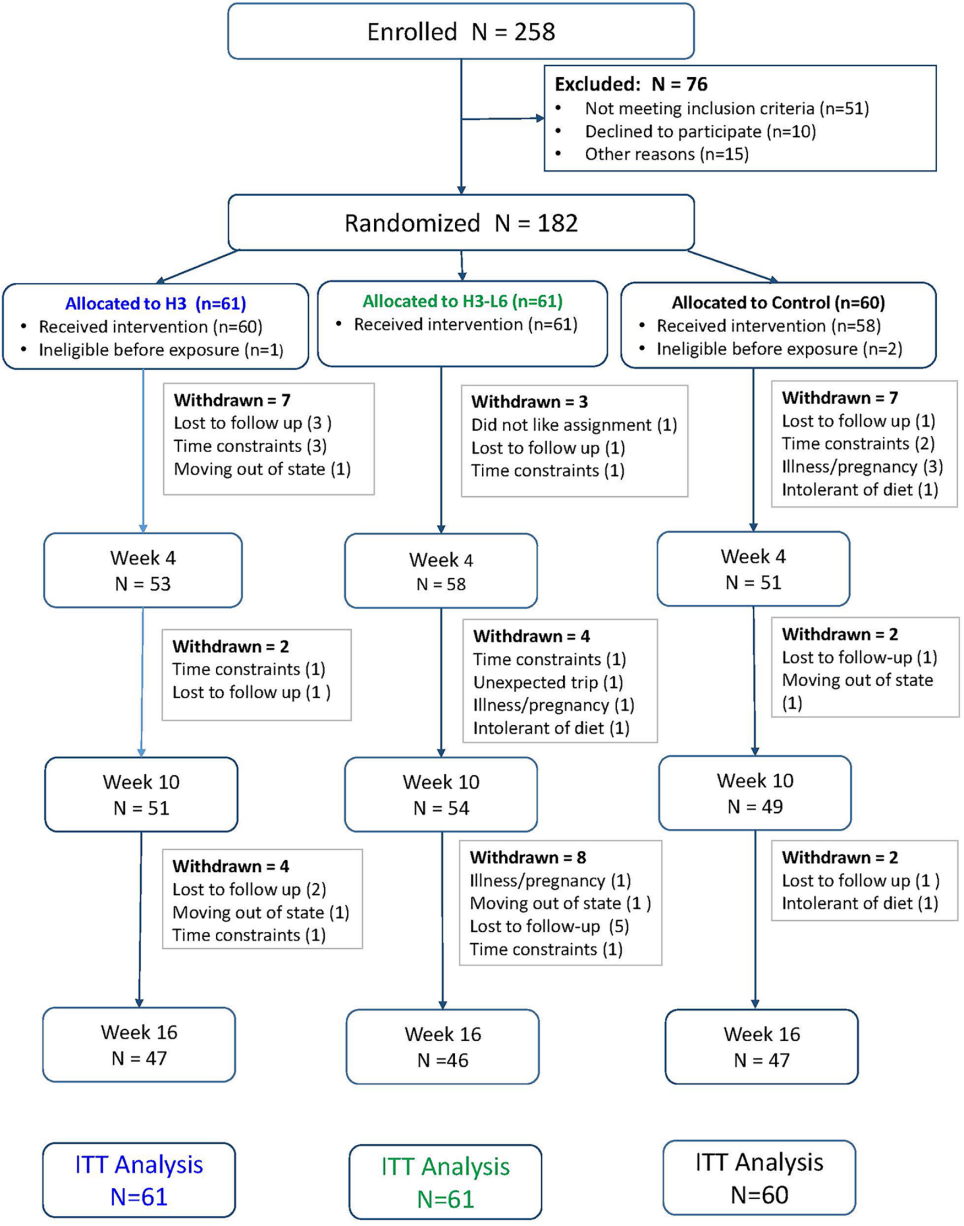
Study flow diagram over the 16 weeks of the trial.

One hundred eighty-two individuals with episodic (*n* = 60) or chronic (*n* = 122) migraine were randomized into the three diet groups with 61 participants each in the intervention groups and 60 participants in the control group (Figure 1). The sample was comparable at baseline. Participants were 18–70 years old. The mean age across the groups ranged from 36.9 in the control group to 39.4 in the H3L6 group with an overall mean of 38.3. Mean body mass index was 29.4. Over 88% of the sample were women and 76% were White. Most (66%) were living with a partner. Over 77% had a college education and the majority had household incomes of more than $40,000 per year. The mean HIT-6 at baseline was in the severely affected range (>60) and the mean MIDAS score was consistent with severe disability in all three groups. Participants had a mean of 5.4 headache hours per day with an average of 16.2 headache days per month along with an average number of severe headache days per month of 2.0. At baseline, participants were taking several preventative and adjunctive medications, including Botulinum toxin, anticonvulsants, muscle relaxers, antidepressants, anxiolytics, and beta blockers. The credibility of the interventions was somewhat higher for the diets that increased omega-3 compared to the control (Table 1).

**Table 1:** Characteristics of the study population.

	H3		H3L6		Control	
	(*n* = 61)		(*n* = 61)		(*n* = 60)	
Demographic characteristics	Mean or *N*	SD or %	Mean or *N*	SD or %	Mean or *N*	SD or %
Age in years	38.8	(11.9)	39.4	(11.7)	36.9	(12.5)
Female	52	(85%)	57	(93%)	52	(87%)
Race^a^						
White	45	(76%)	46	(77%)	47	(78%)
Black or African American	8	(14%)	14	(23%)	11	(18%)
Asian	3	(5%)	0	(0%)	0	(0%)
More than one race	3	(5%)	0	(0%)	2	(3%)
Partnered relationship	39	(65%)	38	(63%)	42	(71%)
Education level						
Some college or less	14	(23%)	18	(30%)	10	(17%)
Associate's or bachelor's degree	25	(41%)	24	(40%)	31	(53%)
Post graduate	22	(36%)	18	(30%)	18	(31%)
Annual household income <$40,000	25	(42%)	16	(28%)	28	(47%)
Baseline clinical characteristics						
Headache impact test	62.7	(5.6)	63.2	(4.7)	62.3	(5.7)
Headache hours per day^b^	5.7	(4.3)	5.5	(4.0)	5.1	(4.1)
Severe headache hours per day^b^	2.1	(2.1)	2.0	(1.8)	2.0	(2.1)
Headache days per month^b^	16.5	(6.2)	16.0	(6.4)	16.3	(6.3)
Meets ICDH-3 criteria for chronic migraine	38	(62%)	42	(69%)	42	(70%)
Migraine with aura	14	(23%)	19	(31%)	18	(30%)
PROMIS-29						
Physical function	45.9	(9.3)	48.5	(8.6)	47.3	(9.0)
Anxiety	52.0	(8.8)	51.7	(9.4)	51.0	(8.5)
Depression	47.1	(7.2)	46.5	(7.2)	46.6	(6.8)
Fatigue	53.6	(9.7)	55.5	(8.3)	53.9	(10.9)
Sleep disturbance	51.5	(5.7)	51.5	(4.7)	51.4	(5.4)
Pain interference	59.2	(6.5)	58.3	(6.9)	59.6	(6.6)
Social role function	47.7	(7.2)	46.8	(8.2)	47.7	(8.4)
Pain intensity (0–10)	4.5	(2.2)	4.3	(1.9)	4.5	(1.9)
Percentage of time with pain	50%	(29)	47%	(30)	44%	(27)
Perceived benefit (Headache) (5-1)^c^	3.0	0.6	3.1	0.5	3.0	0.6
Perceived benefit (Overall) (5-1)^c^	3.0	0.5	3.0	0.4	3.0	0.3
Perceived satisfaction with care (1–5)^c^	3.4	0.8	3.5	0.8	3.2	1.0
MIDAS (median, IQR)	31	(12, 49)	31	(16, 51)	22	(10, 48)
Overall health (1–4)	2.75	(0.44)	2.73	(0.43)	2.71	(0.46)
Sleep quality (1–4)	2.55	(0.48)	2.49	(0.48)	2.53	(0.54)
Stress level (0–10)	2.98	(1.65)	2.71	(1.48)	3.29	(1.65)
Whole Body Pain Scale						
Number of painful sites	7.21	(4.59)	7.46	(4.98)	6.68	(4.51)
Percentage of time with pain	5.02	(2.92)	4.74	(2.99)	4.37	(2.74)
Preventive headache pain medications	1.1	(1.4)	1.3	(1.5)	0.9	(1.1)
Botulinum toxin	5	(8%)	7	(11%)	2	(3%)
Anticonvulsants	19	(31%)	24	(39%)	5	(8%)
Muscle relaxers	8	(13%)	9	(15%)	4	(7%)
Antidepressants	18	(30%)	17	(28%)	25	(42%)
Sedatives	6	(10%)	6	(10%)	6	(10%)
Beta blockers or verapamil	8	(13%)	13	(21%)	7	(12%)
Body mass index	29.1	(8.4)	29.8	(11.3)	29.3	(7.3)
Credibility of intervention group^d^	35.9	(7.4)	35.2	(8.3)	32.2	(7.7)

^a^ Three declined to answer.

^b^ Derived from the daily diary.

^c^ Standard errors are presented.

^d^ Based on the Borkovec and Nau credibility questionnaire.

### 3.2. Changes in daily stress, sleep quality, and perceived health

**Figure 2:**
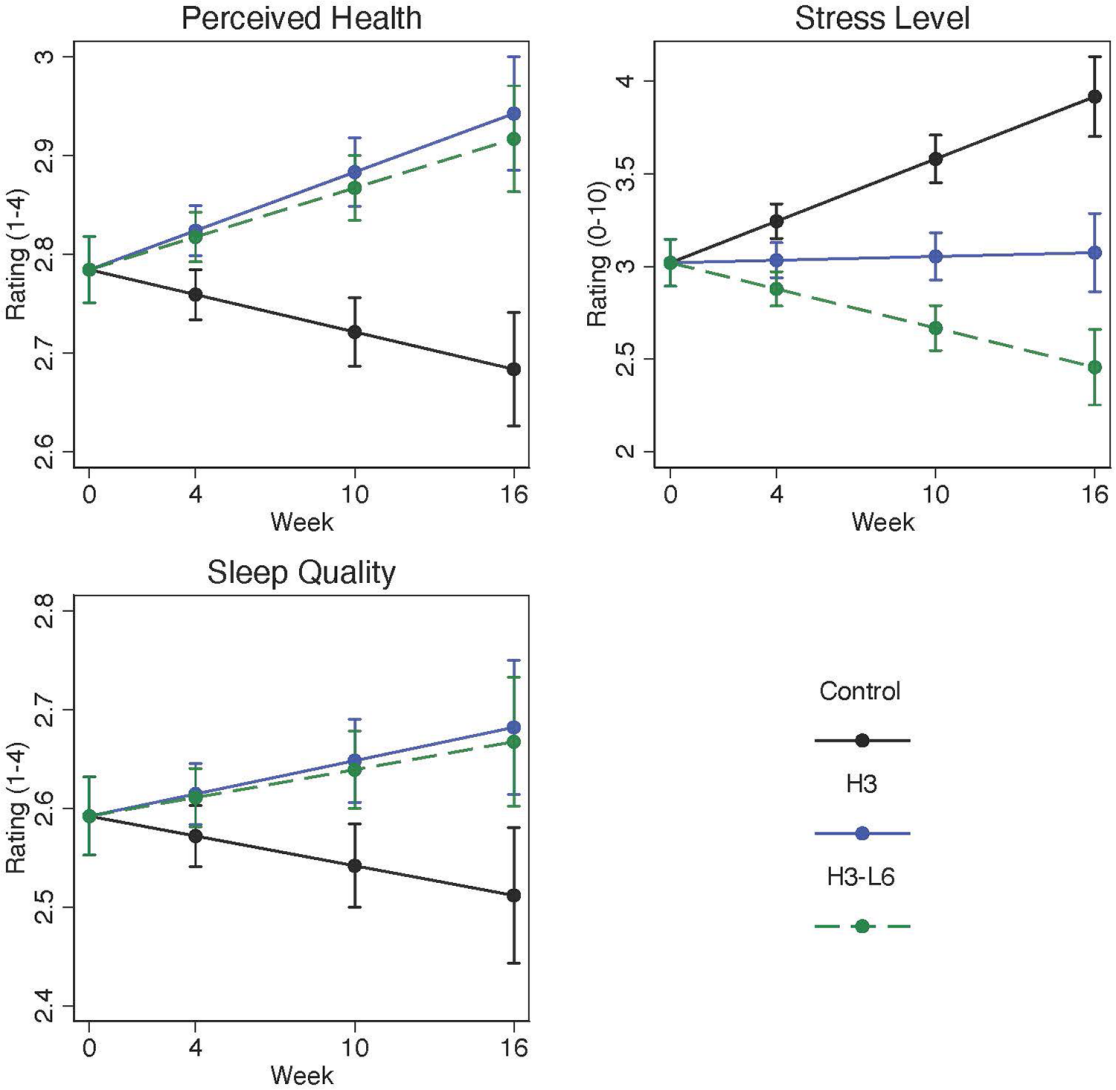
Change in daily perceived health, stress, and sleep quality over 16 weeks. Plots are model-predicted means and 95% confidence intervals for each group at each of the study visits. Linear population-averaged mixed effects models were constructed using an autoregressive correlation using daily diary measurements of each endpoint regressed on group-by-time interaction, time, and recruitment site.

Perceived stress, based on daily estimates, was lower in the intervention groups at the end of the trial compared with the control group. On a 0–10 scale, the post-intervention adjusted mean was 3.9 [95% Confidence Interval (CI): 3.7–4.1] in the control group compared with 2.5 (95% CI: 2.3–2.7) in the H3L6 group and 3.1 (95% CI: 2.9–3.3) in the H3 group representing large effect sizes as measured by Cohen's d. Sleep quality improved significantly in the intervention groups compared with the control group with moderate effect sizes. At baseline, the overall sleep quality was 2.5 (SD 0.49) on a 1–4 scale. Adjusted means at intervention end were greater for the H3L6 and H3 intervention groups: H3L6 2.7 (2.6–2.7) and H3 2.7 (2.6–2.8). Perceived overall health also improved significantly in the intervention groups compared with the control group with large effect sizes (Table 2 and Figure 2).

**Table 2:** Adjusted mean difference in endpoints at intervention end^a^.

	H3 vs control		H3L6 vs control	
	Mean difference (95% CI)	Cohen's d	Mean difference (95% CI)	Cohen's d
PROMIS-29^b^				
Pain intensity	−0.6 (−1.4 to 0.1)	−0.30	−0.6 (−1.3 to 0.1)	−0.31
Pain interference	−3.2 (−5.9 to −0.5)	−0.42	−1.8 (−4.4 to 0.7)	−0.25
Fatigue	−2.0 (−5.4 to 1.3)	−0.21	−2.9 (−6.2 to 0.5)	−0.28
Anxiety/fear	−0.7 (−3.8 to 2.4)	−0.08	−3.5 (−6.6 to −0.4)	−0.39
Depression/sadness	0.9 (−1.5 to 3.4)	0.13	−0.4 (−2.9 to 2.2)	−0.05
Sleep disturbance	0.2 (−1.8 to 2.2)	0.04	−0.01 (−1.9 to 1.9)	−0.00
Social roles/activities	1.9 (−0.9 to 4.7)	0.23	0.5 (−2.5 to 3.4)	0.06
Physical function	1.4 (−1.4 to 4.2)	0.17	1.2 (−1.5 to 4.0)	0.15
MIDAS	−10.7 (−24.0 to 2.7)	−0.26	−11.8 (−25.1 to 1.5)	−0.30
Diary Measures^c^				
Overall health (1–4)	0.3 (0.2–0.3)	1.14	0.2 (0.2–0.3)	1.06
Sleep quality (1–4)	0.2 (0.1–0.3)	0.63	0.2 (0.1–0.2)	0.59
Stress (0–10)	−0.8 (−1.1 to −0.5)	−0.99	−1.5 (−1.7 to −1.2)	−1.76
Whole-Body Pain Scale				
Number of painful sites^d^	−0.1 (−0.3 to 0.1)	−0.17	−0.04 (−0.2 to 0.2)	−0.05
Percent of time with pain^e^	−1.6 (−2.5 to −0.6)	−0.55	−0.4 (−1.4 to 0.6)	−0.15
Perceived benefit (overall)ᶠ	−0.2 (−0.5 to 0.1)	−0.27	−0.1 (−0.4 to 0.2)	−0.15
Perceived benefit (headache)ᶠ	−0.3 (−0.6 to 0)	−0.36	−0.4 (−0.7 to −0.1)	−0.43
Perceived satisfaction with careᶠ	0.1 (−0.2 to 0.4)	0.12	0.02 (−0.3 to 0.4)	0.02

^a^ All values control for baseline values and site.

^b^ Pain Intensity is measured on a 0–10 scale. The remain measures are *T*-scores: population average is 50 with a standard deviation of 10.

^c^ Based on daily diary: Sleep quality and perceived health were measured on a 1–4 scale with 4 indicating better outcomes. Stress was measured on a 0–10 scale. 10 = most stress.

^d^ Poisson regression was performed for the number of painful sites. Group differences are in ratios.

^e^ Based on a single Likert scale (0–10) question. Each value represents 10% pain, with 10 = 100% pain.

^f^ Each question is a 1–5 scale with 5 = Much Worse for perceived benefit (overall and headache) and 5 = Very Satisfied for satisfaction with care.

### 3.3. Changes in the PROMIS-29

Of the PROMIS-29 measures, only pain interference, with a mean of 59.0 [Standard Deviation (SD) 6.6] was substantially elevated at baseline (Table 1: Baseline Characteristics). The pain interference score at baseline corresponded to a G-code severity assessment of 40%–59% impaired (24). The baseline physical function score of 47.3 (SD 9.0) and the PROMIS fatigue score of 54.3 (SD 9.6) corresponded to a G-code impairment of 20%–39%. The mean pain intensity was 4.4 (SD 2.0) on a 0–10 scale. Scores for mean anxiety, depression, and sleep disturbance were not elevated at baseline. Measures of social role function were not meaningfully different from population norms.

**Figure 3:**
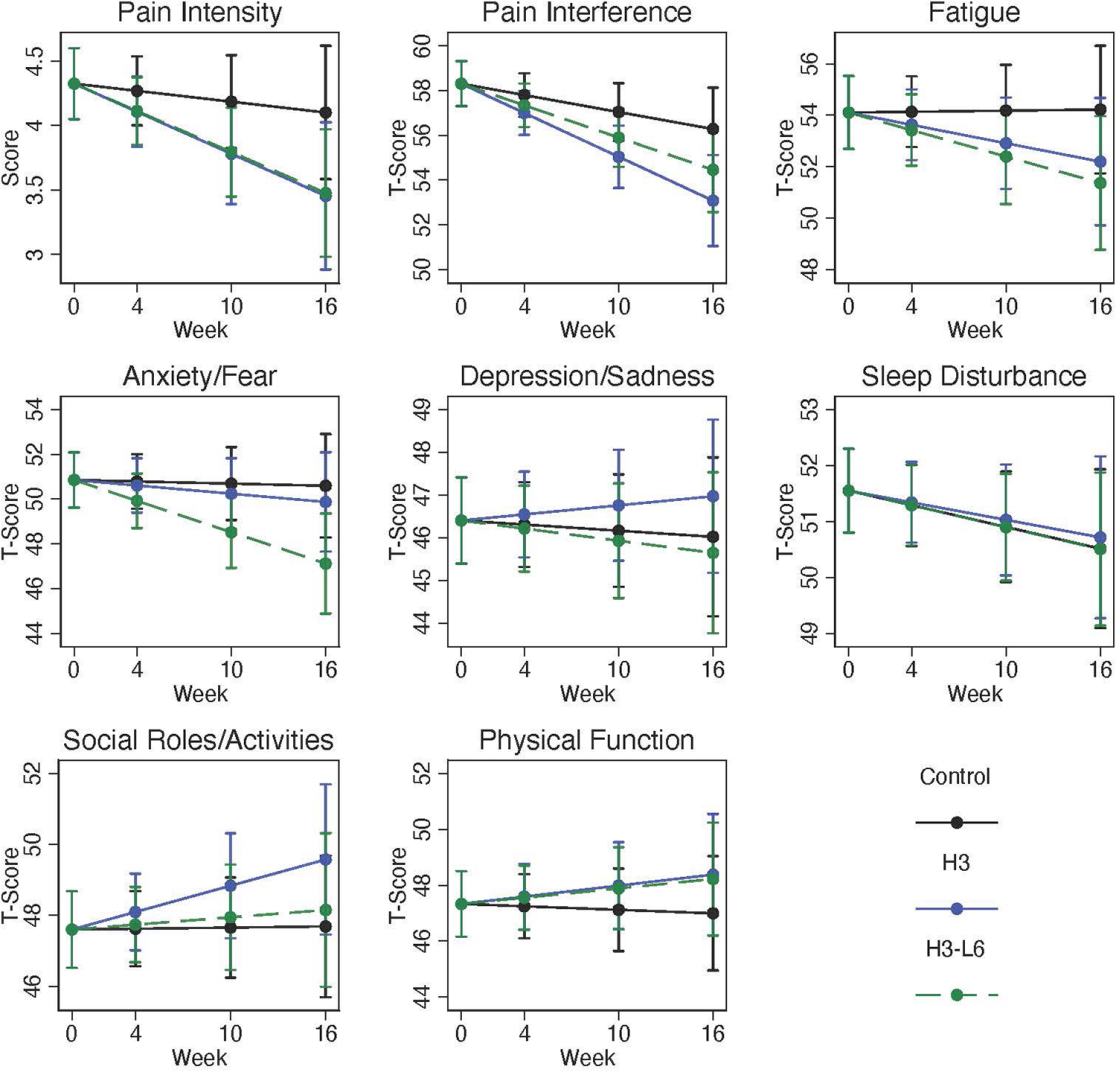
Change in PROMIS-29 domains over 16 weeks. Plots are model-predicted means and 95% confidence intervals based on generalized linear models with each outcome regressed on the group-by-time interaction, time, and recruitment site.

Self-reported pain interference improved within both diet intervention groups (Figure 3). The H3 diet resulted in a pain interference score at 16 weeks 3.2 points lower in the H3 compared with the control group (95% CI: −5.9, −0.5) and 1.8 points lower in the H3L6 diet group compared with the control group (95% CI: −4.4, 0.7). Pain intensity improved by 19% and 22% in the two intervention groups and by 9% in the Control group. The post-intervention differences in pain intensity favored the intervention groups with a change in the post-intervention (0–10) pain scale of −0.6 (−1.4, 0.1) in the H3 group compared with the control and −0.6 (−1.3, 0.1) in the H3L6 group.

Small increases in depression scores occurred in all three groups; these were not significantly different between groups. Similarly, anxiety and social role function levels were close to the population average (50) at baseline and changed little over the course of the trial. Based on the PROMIS measures, sleep disturbance was slightly higher than the general population at baseline and changed very little over the course of the 16-week intervention.

### 3.4. Changes in migraine disability

MIDAS scores dropped significantly within each group (Table 2), with greater reductions in the intervention groups. At 16 weeks, the median score dropped from 28 to 12, 31 to 12, and 22 to 17 in the H3, H3L6, and control groups, respectively. The between-group differences in the means at 16 weeks, were not statistically significant (*p* = 0.1 and 0.08 for the H3-vs.-control and H3L6-vs.-control comparisons, respectively).

### 3.5. Changes in perceived benefits and satisfaction with care

Perceived benefits of the diets for overall health were reported in all three groups, without significant between-group differences. Similarly, satisfaction with care improved in all three groups. Consistent with the change in headache hours per day, perceived benefit to the headache condition was greater in the intervention groups than in the control group.

### 3.6. Changes in the whole-body pain scale

At baseline, the average number of painful body sites at baseline across the sample was 7.1 (SD 4.7). The average percentage of time participants said they had at least some pain was 47.1% (SD 28.8). The number of painful body sites was comparable across the three groups and changed little over time, but the percentage of time that individuals experienced pain changed by the end of the trial by −42% in the H3 diet group, −14% in the H3L6 diet group, and 3% in the Control group. The differences between groups favored the intervention groups but were not statistically significant.

### 3.7. Heterogeneity assessments

In the sensitivity analysis, no clear pattern emerged in the intervention effects comparing chronic migraine (*n* = 122) to episodic migraine (*n* = 60) (Supplementary Table S1). The MIDAS disability score changed more in the intervention groups relative to the control for participants with chronic migraine relative to those with episodic migraine but the differences were not statistically significant.

Participants were classified as having migraine with aura if at least some of their migraine attacks included an aura. Comparing migraine with aura (*n* = 51) to migraine without aura (*n* = 131) revealed greater improvements in the diary measures (perceived health, sleep quality, stress) for the intervention groups compared with the control among participants with migraine with aura (Supplementary Table S2). For example, perceived stress was 2.1 points lower (95% CI: 1.6, 2.7) comparing the H3 group (*n* = 14) to the control (*n* = 18) among participants with aura whereas a reduction of 0.3 (95% CI: 0.01, 0.7) was seen comparing the H3 group (*n* = 47) to the control (*n* = 42) among participants without aura. Similarly, in the H3L6 group (*n* = 19) relative to the control (*n* = 18), a reduction of 2.3 points (1.8, 2.8) was seen among patients with aura and a reduction of 1.1 points (95% CI: 0.8, 1.4) comparing the H3L6 group (*n* = 42) to the control (*n* = 42) was seen among participants without aura.

### 3.8. Changes in plasma fatty acids

As expected, the average increase in EPA and DHA in plasma was 84% and 90% respectively in the H3 diet group. Increases in EPA and DHA in the H3L6 group were 63% and 56%; both groups exhibited substantial increases as compared with the control group. The H3L6 diet was also designed to decrease LA in plasma. Decreases in LA were modest at 8% with a 10% reduction in AA (Table 3).

**Table 3:** Changes in plasma fatty acid concentrations (µg/ml), *n* = 151.

	Values at baseline and week 16				Differences between groups at week 16^a^			
		H3 (*n* = 50)	H3L6 (*n* = 53)	Control (*n* = 48)		H3 vs.	H3L6 vs.	H3L6 vs.
		Median (IQR)	Median (IQR)	Median (IQR)		Control	Control	H3
Omega−3 fatty acids								
EPA + DHA	Baseline	42 (34, 53)	50 (36, 58)	49 (36, 58)				
	Week 16	77 (51, 113)	76 (57, 112)	47 (35, 60)	Difference	43 (31, 56)	37 (25, 49)	−6.1 (−18, 5.8)
	% Change	+86%	+53%	−4%	*p*-value	<0.001	<0.001	0.31
EPA (20:5n3)	Baseline	10 (6.5, 14)	12 (7.8, 14)	10 (7.0, 15)				
	Week 16	19 (12, 32)	19 (13, 31)	11 (8.1, 16)	Difference	12 (7.6, 16)	11 (6.2, 15)	−1.4 (−5.8, 3.0)
	% Change	+84%	+63%	+2%	*p*-value	<0.001	<0.001	0.52
DHA (22:6n3)	Baseline	33 (27, 39)	36 (27, 44)	37 (27, 45)				
	Week 16	62 (43, 82)	56 (45, 81)	35 (28, 45)	Difference	31 (23, 39)	27 (19, 35)	−4.6 (−13, 3.4)
	% Change	+90%	+56%	−4%	*p*-value	<0.001	<0.001	0.26
Omega−6 fatty acids								
LA (18:2n6)	Baseline	749 (653, 838)	745 (664, 896)	764 (676, 901)				
	Week 16	758 (678, 881)	686 (579, 867)	760 (583, 873)	Difference	37 (−27, 101)	−3.1 (−66, 60)	−40 (−103, 23)
	% Change	+1%	−8%	−1%	*p*-value	0.26	0.92	0.21
AA (20:4n6)	Baseline	173 (143, 214)	204 (170, 227)	176 (151, 236)				
	Week 16	147 (124, 209)	183 (159, 214)	180 (147, 233)	Difference	−17 (−32, −1.5)	−13 (−28, 2.0)	3.7 (−11, 19)
	% Change	−15%	−10%	+2%	*p*-value	0.03	0.09	0.62

EPA, eicosapentaenoic acid; DHA, docosahexaenoic acid; LA, linoleic acid; AA, arachidonic acid.

^a^ Baseline-adjusted values are based on ANCOVA controlling for the recruitment site.

**Figure 4:**
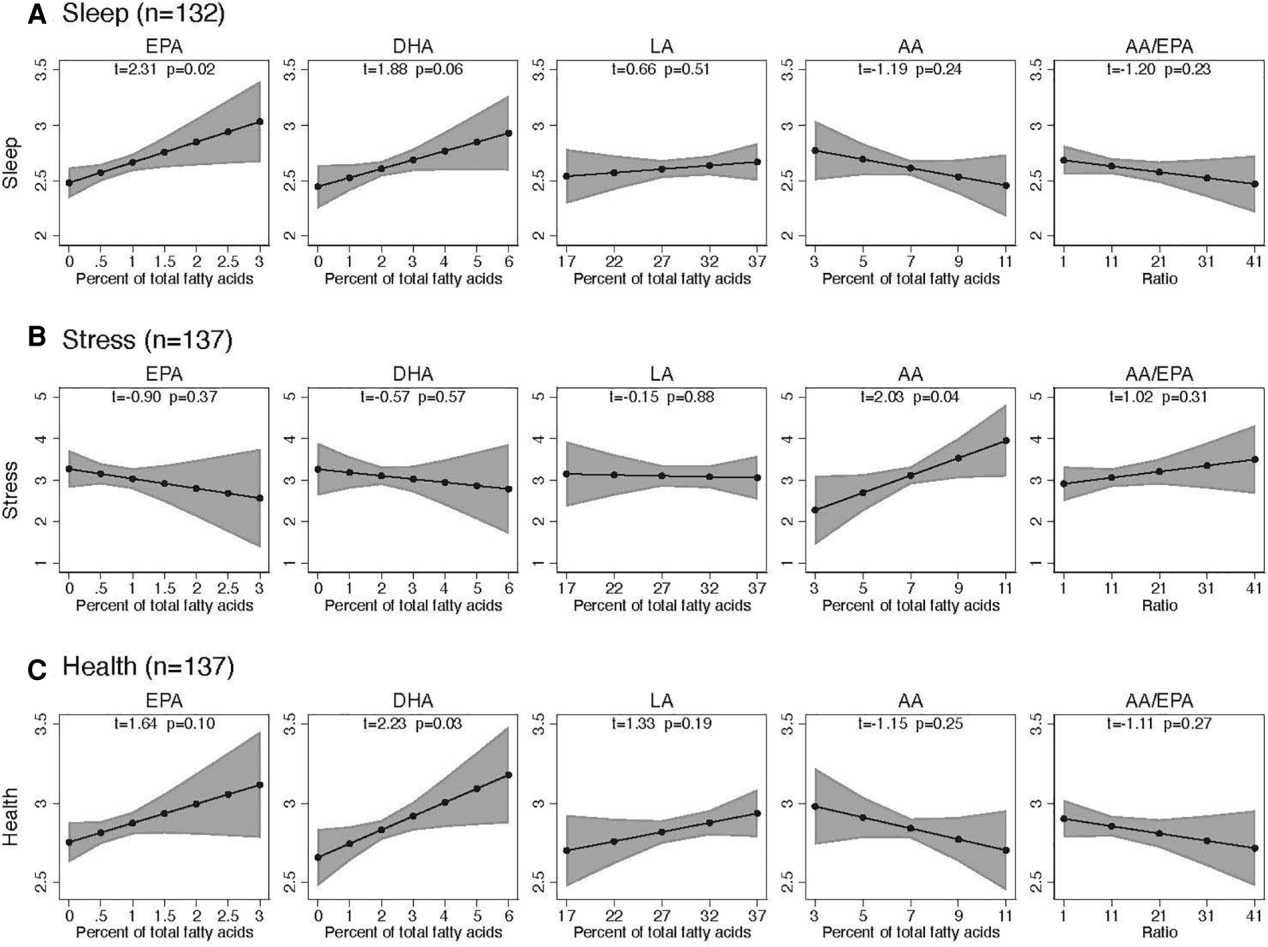
Association of sleep, stress, and health with precursor fatty acids in plasma over 16 weeks. Each plot stands for coefficients and 95% confidence intervals from a linear regression model adjusted for the following baseline variables: respective endpoint, respective fatty acid, age, BMI, sex, headache days per month, HIT-6, and chronic-vs.-episodic migraine.

Increases in omega-3 EPA were associated with improved sleep quality (*t* = 2.31, *p* = 0.02) and a trend in improved perceived health at the end of the trial (*t* = 1.64, *p* = 0.10). Increases in omega-3 DHA were associated with improved perceived health (*t* = 2.23, *n* = 0.03) and a trend in improved sleep quality (*t* = 1.88, *p* = 0.06). Changes in omega-6 LA were not significantly associated with sleep quality, perceived stress, or perceived health. Perceived stress increased with increasing omega-6 AA (*t* = 2.03, *p* = 0.04). (Figure 4).

### 3.9. Adverse events

Adverse event rates overall did not differ among the intervention groups: 47% in the control diet group, 38% in the H3 diet group, and 49% in the H3L6 diet group. Most (93%) were mild and most (81%) were not related or possibly related to any aspect of the interventions (10). Phlebotomy-related events occurred in all three groups (bruising, vasovagal reaction). Of the remaining six adverse events reported in the H3L6 diet group, three were associated with gastrointestinal complaints (nausea, dyspepsia), two with a rash or itching, two with weight loss, and one with a worsening headache. In the H3 diet group, one participant reported a gastrointestinal symptom, and one reported a rash. In the control diet group, one participant reported a worsening headache.

## 4. Discussion

### 4.1. Summary

In this 3-arm trial that randomized 182 adults with chronic/episodic migraine to an H3 diet, H3L6 diet or a control diet, at baseline, participants had over 16 mean headache days per month and a HIT-6 mean score in the severe impact range. This analysis focused on the prespecified secondary trial endpoints including daily diary measures (stress, perceived health, and sleep quality), non-headache pain and disability measures (PROMIS pain intensity and pain interference, MIDAS), and other symptoms that co-occur with headache (PROMIS anxiety, depression, sleep quality, fatigue, social role satisfaction). Briefly, at the end of the trial, stress, perceived health, and sleep quality improved significantly in the intervention diet groups relative to the control diet group and these changes were stronger among participants who reported migraines with aura. Pain interference, non-headache pain, and pain-related disability also improved meaningfully in the intervention diet groups relative the control, but the difference was not statistically significant. We saw a meaningful and statistically significant decrease in anxiety in the H3L6 diet group relative to the control but not in the H3 diet group. Marked increases in omega-3 fatty acids in the intervention diet groups is consistent with increased intakes. Linoleic acid in plasma was modestly reduced.

### 4.2. Daily measures of perceived health, sleep quality, and stress

At baseline, as measured in the diary, perceived health and sleep quality were less than 3 (good) on a scale ranging from 1 (poor) to 4 (excellent) and perceived stress was not elevated. Over the course of the trial, all three daily measures improved significantly more in the H3 and H3L6 intervention groups compared with the control intervention, with large effect sizes. Changes in omega-3 plasma fatty acid concentrations were associated with improvements in sleep and perceived health. Changes in arachidonic acid were associated with changes in stress.

These results suggest that a dietary intervention increasing dietary omega-3 fatty acids with and without reduction in omega-6 linoleic acid can improve sleep, stress, and perceived health among people with chronic/episodic migraine. While it is not clear whether the diets have a direct effect on these comorbid symptoms, or whether they improve due to pain reductions, the association between higher omega-3 fatty acid levels and improved sleep and perceived health provide a mechanistic clue.

In the literature, little data are available examining the effect of omega-3 intakes on sleep in adults. A cross-sectional study examined the association between omega-3 intakes and diagnosed sleep disorders in the National Health and Nutrition Examination Survey (NHANES) 2007–2014 (29). Compared with the lowest tertile, the highest tertile of omega-3 intakes was associated with a lower prevalence of sleep disorders [adjusted odds ratio (aOR) 0.85; CI 0.70–1.03] and the highest omega-6-to-omega-3 ratio was associated with a higher prevalence of sleep disorders (aOR 1.36; CI 1.08–1.70) (29). Another study based on NHANES 2011–2012 examined the association between serum fatty acid levels and sleep parameters (30). In multivariable models, very short (<5 h) and short (5–6 h) sleep durations were associate with lower mean omega-3% of fatty acid levels (adjusted mean difference: −34%; 95% CI −0.58, −0.11 for very short and −0.10%; −0.28, 0.08 for short) (30).

Data on the association between perceived stress and arachidonic acid (AA) levels is also sparse. In a study of motor vehicle accident survivors, higher baseline levels of both AA and EPA were associated with a lower risk of posttraumatic stress disorder) (31).

### 4.3. General pain and pain-related disability

Baseline levels of (PROMIS) pain interference were consistent with 40%–59% impairment and the average pain intensity was in the moderate range. Baseline MIDAS scores were also consistent with a high degree of disability. In this analysis, we showed that pain interference improved to a greater extent in the intervention groups. Similarly, average pain intensity was lower in the intervention groups at the end of the trial. Controlling for baseline levels, MIDAS disability scores were also clinically meaningfully improved in the intervention groups compared to the control group (although not statistically significant). These findings support the hypotheses that those with high pain and pain-related disability at baseline improved when assigned to dietary omega-3 fatty acid diets. Moreover, these findings are consistent with our previous findings that increases in omega-3 fatty acids are associated with improvements in pain (10).

### 4.4. Comorbid physical and psychological symptoms

Surprisingly, mean anxiety, sleep disturbance, and mean depression scores were at or below population norms despite high baseline levels of disability as evidenced by the MIDAS and Headache Impact Test. This may be explained in part by the high proportion of individuals taking antidepressants at baseline (33%). Unsurprisingly, these measures changed little over the course of the intervention and between-group differences were small. In addition, baseline levels of fatigue, physical function, and social role functioning were not meaningfully abnormal. These findings contradict those in the literature—depression, anxiety, and sleep disorders often co-occur with migraine (32–34) and, based on the baseline headache history, sleep disturbance was diagnosed in 30% of participants.

One possible explanation for the lack of abnormality in these symptoms is the measurement instrument. PROMIS measures were developed and validated in a general US population and may not be sensitive enough to phenotype chronic pain populations. In a large cross-sectional study in an orthopedic population with chronic pain, depression, anxiety, fatigue, and sleep disturbance were measured at levels similar to our study (35). In another large (*n* = 750) study of chronic low back pain patients, PROMIS anxiety and depression levels were measured at <50 (36). In two large studies of orthopedic patients, the PROMIS depression score showed a strong floor effect, bringing the validity of the measurement into question (37, 38) Another possible explanation is that individuals with chronic pain did not consider their depression/anxiety levels to be abnormal.

Unlike the sparse data on the association between sleep and fatty acids, the impact of essential dietary fatty acids on depression/anxiety has been an active area of research with 17 ongoing studies of omega-3 intakes vs. placebo for depression reported in a recent Cochrane review (39). Despite the interest, definitive conclusions have been elusive. For example, a meta-analysis of 33 studies of omega-3 intakes in varying doses (from food or supplements) reported a small reduction in depressive symptomatology (−0.40, 95% CI: −0.64, −0.16) albeit with a high degree of heterogeneity in sample populations, omega-3 sources, trial durations, and outcome measures. In addition, risk of bias was high in some studies and omega-6 intakes were not reported. A second 2021 systematic review and meta-analysis investigated the effects of high vs. lower omega-3 intakes, mostly from supplements, on the development of anxiety or depression (40). Intakes of omega-6 were not considered although one trial examined total PUFA intakes. The authors concluded that higher omega-3 intakes are not associated with a lower risk of depression [Risk Ratio (RR) 1.01; 95% CI: 0.92, 1.10]. Fewer studies examined anxiety outcomes. Results suggested that higher omega-3 slightly increased anxiety symptoms (standardized mean difference: 0.15; 95% CI 0.05–0.26), but the quality of included studies was poor.

### 4.5. Study limitations and strengths

Well-documented challenges of conducting dietary intervention trials include difficulties with blinding, high attrition, and limited compliance (41, 42). In this three-arm trial in a free-living migraine population, blinding was achieved using a credible control diet. Attrition was higher than desired, at 23%, but 85% remained in the trial for at least 10 weeks. Dietary adherence was acceptable: omega-3 *intakes* increased by greater than 2900% in both intervention diets and omega-6 decreased by 49% in the H3L6 diet (24). With these dietary adjustments, we saw significant and meaningful changes in daily assessments of sleep quality and perceived health, but fewer changes in recalled self-reported measures compared to the Chronic Daily Headache study.

A strength of the study is its use of an electronic headache diary that captured details of the headache experience, including associated symptoms. For example, the diary assessed not only pain at each hour of the day, but also sleep quality, perceived health, stress levels, and acute medication taken for pain. The diary was completed daily, with individualized prompts for non-completion leading to an overall completion rate of >80% throughout the active intervention. Participants had to complete the diary within 48 h which vastly reduced the potential for recall bias common to intermittent questionnaires and paper diary measures. In fact, in a comparison of the diary with recalled measures of headache frequency and severity, more headaches were reported in the diary (43). Other studies also suggest that an electronic headache diary may be superior to recall measures (44).

### 4.6. Future research

The value of omega-3 and omega-6 manipulations in the diet for health outcomes clearly is still an open question. The importance of precision nutrition in the heterogeneity of responses to dietary interventions has been recognized in recent years with a steady increase in precision nutrition studies (45). Unfortunately, changes in the tissue levels of fatty acids and their derivatives that could be responsible for changes in clinical outcomes take weeks to months rather than days, making short-term feeding studies untenable. We continue to feel that this line of research is critical due to the burden of migraine, incomplete responses to migraine medications, side effects associated with medication use, and quality-of-life deficits associated with migraine. We plan to continue this line of research to understand the impact of omega-3 and omega-6 metabolites on migraine and their psychological correlates.

## 5. Conclusions

In this study of a whole-foods dietary intervention for the prevention of migraine headaches, the H3 and H3L6 intervention diets resulted in greater improvements in perceived stress, perceived health, and sleep quality compared to the control diet. Further research is needed to prove the value of these interventions for the management of chronic pain and comorbid symptoms in the overall population of individuals with chronic pain.

## Data Availability

The data analyzed in this study is subject to the following licenses/restrictions: De-identified data may be requested from the corresponding author subject to a data use agreement with the University of North Carolina at Chapel Hill. Requests to access these datasets should be directed to faurot@med.unc.edu.
