# Supplementary material for: Dietary fatty acids improve perceived sleep quality, stress, and health in migraine: a secondary analysis of a randomized controlled trial
Source: Front Pain Res (Lausanne). 2023 Oct 25;4:1231054. doi: 10.3389/fpain.2023.1231054 (PMC10634433; doi:10.3389/fpain.2023.1231054)
Supplement: Supplementary file 1 [file Table1.docx]

**Table S1. Subgroup (heterogeneity) analysis by baseline chronic/episodic migraine ᵃ**

|  |  | **Episodic migraine (n=60)** | | |  | **Chronic migraine (n=122)** | | |
| --- | --- | --- | --- | --- | --- | --- | --- | --- |
|  |  | **Control (n=18)** | **H3 (n=19)** | **H3L6 (n=23)** |  | **Control (n=42)** | **H3 (n=42)** | **H3L6 (n=38)** |
| **PROMIS-29 ᵇ** | | | | | | | | |
| Pain intensity | **Estimate (95% CI)** | 4.7 (3.7 to 5.8) | 3.6 (2.6 to 4.6) | 4.7 (3.9 to 5.5) |  | 5.3 (4.7 to 5.8) | 4.8 (4.2 to 5.5) | 4.3 (3.7 to 4.9) |
|  |  |  | *Versus Control* | |  |  | *Versus Control* | |
|  | **Difference (95% CI)** |  | -1.1 (-2.6 to 0.3) | -0.03 (-1.3 to 1.3) |  |  | -0.4 (-1.3 to 0.4) | -1.0 (-1.8 to -0.1) |
|  | **p-value** |  | 0.13 | 0.96 |  |  | 0.31 | 0.02 |
|  | **Cohen's d** |  | -0.28 | -0.01 |  |  | -0.18 | -0.41 |
|  |  |  |  |  |  |  |  |  |
| Pain interference | **Estimate (95% CI)** | 55.2 (51.6 to 58.8) | 49.0 (45.5 to 52.6) | 54.8 (51.4 to 58.1) |  | 56.8 (54.8 to 58.9) | 55.0 (52.7 to 57.2) | 54.1 (52.0 to 56.3) |
|  |  |  | *Versus Control* | |  |  | *Versus Control* | |
|  | **Difference (95% CI)** |  | -6.2 (-11.1 to -1.2) | -0.4 (-5.2 to 4.4) |  |  | -1.9 (-4.9 to 1.1) | -2.7 (-5.6 to 0.2) |
|  | **p-value** |  | 0.01 | 0.87 |  |  | 0.22 | 0.06 |
|  | **Cohen's d** |  | -0.43 | -0.03 |  |  | -0.22 | -0.33 |
|  |  |  |  |  |  |  |  |  |
| Fatigue | **Estimate (95% CI)** | 52.8 (47.9 to 57.7) | 52.0 (47.9 to 56.1) | 51.2 (47.1 to 55.4) |  | 55.0 (52.1 to 57.8) | 52.2 (49.2 to 55.3) | 51.4 (48.3 to 54.5) |
|  |  |  | *Versus Control* | |  |  | *Versus Control* | |
|  | **Difference (95% CI)** |  | -0.8 (-7.1 to 5.6) | -1.5 (-7.7 to 4.6) |  |  | -2.7 (-6.5 to 1.1) | -3.6 (-7.4 to 0.3) |
|  | **p-value** |  | 0.81 | 0.62 |  |  | 0.17 | 0.07 |
|  | **Cohen's d** |  | -0.04 | -0.09 |  |  | -0.23 | -0.30 |
|  |  |  |  |  |  |  |  |  |
| Anxiety/fear | **Estimate (95% CI)** | 51.2 (46.4 to 56.1) | 49.7 (45.8 to 53.6) | 47.6 (44.0 to 51.2) |  | 50.3 (47.9 to 52.7) | 50.0 (47.4 to 52.5) | 46.8 (44.1 to 49.6) |
|  |  |  | *Versus Control* | |  |  | *Versus Control* | |
|  | **Difference (95% CI)** |  | -1.5 (-7.6 to 4.5) | -3.6 (-9.4 to 2.2) |  |  | -0.4 (-3.7 to 3.0) | -3.5 (-6.9 to 0.02) |
|  | **p-value** |  | 0.62 | 0.22 |  |  | 0.84 | 0.05 |
|  | **Cohen's d** |  | -0.09 | -0.21 |  |  | -0.04 | -0.34 |
|  |  |  |  |  |  |  |  |  |
| Depression/sadness | **Estimate (95% CI)** | 47.1 (43.5 to 50.7) | 47.0 (43.9 to 50.0) | 45.6 (42.5 to 48.7) |  | 45.6 (43.5 to 47.6) | 46.9 (44.8 to 49.1) | 45.7 (43.5 to 48.0) |
|  |  |  | *Versus Control* | |  |  | *Versus Control* | |
|  | **Difference (95% CI)** |  | -0.1 (-4.6 to 4.3) | -1.5 (-6.0 to 3.0) |  |  | 1.4 (-1.4 to 4.1) | 0.2 (-2.7 to 3.0) |
|  | **p-value** |  | 0.95 | 0.51 |  |  | 0.34 | 0.91 |
|  | **Cohen's d** |  | -0.01 | -0.11 |  |  | 0.16 | 0.02 |
|  |  |  |  |  |  |  |  |  |
| Sleep disturbance | **Estimate (95% CI)** | 50.1 (47.0 to 53.3) | 50.3 (47.8 to 52.8) | 50.5 (48.2 to 52.9) |  | 50.7 (49.1 to 52.3) | 50.9 (49.2 to 52.6) | 50.5 (48.8 to 52.2) |
|  |  |  | *Versus Control* | |  |  | *Versus Control* | |
|  | **Difference (95% CI)** |  | 0.2 (-3.7 to 4.1) | 0.4 (-3.4 to 4.3) |  |  | 0.3 (-2.0 to 2.5) | -0.2 (-2.5 to 2.0) |
|  | **p-value** |  | 0.93 | 0.83 |  |  | 0.83 | 0.85 |
|  | **Cohen's d** |  | 0.02 | 0.04 |  |  | 0.04 | -0.03 |
|  |  |  |  |  |  |  |  |  |
| Social roles/activities | **Estimate (95% CI)** | 47.5 (43.6 to 51.5) | 52.1 (48.6 to 55.7) | 48.1 (44.6 to 51.7) |  | 47.7 (45.5 to 49.9) | 48.4 (45.9 to 50.9) | 48.3 (45.8 to 50.8) |
|  |  |  | *Versus Control* | |  |  | *Versus Control* | |
|  | **Difference (95% CI)** |  | 4.6 (-0.6 to 9.8) | 0.6 (-4.6 to 5.8) |  |  | 0.7 (-2.5 to 3.8) | 0.6 (-2.7 to 3.8) |
|  | **p-value** |  | 0.09 | 0.82 |  |  | 0.68 | 0.73 |
|  | **Cohen's d** |  | 0.31 | 0.04 |  |  | 0.07 | 0.06 |
|  |  |  |  |  |  |  |  |  |
| Physical function | **Estimate (95% CI)** | 47.4 (43.0 to 51.8) | 50.6 (47.0 to 54.2) | 49.1 (45.7 to 52.6) |  | 46.7 (44.4 to 49.0) | 47.3 (44.7 to 49.9) | 47.9 (45.4 to 50.3) |
|  |  |  | *Versus Control* | |  |  | *Versus Control* | |
|  | **Difference (95% CI)** |  | 3.2 (-2.5 to 8.8) | 1.7 (-3.5 to 7.0) |  |  | 0.6 (-2.6 to 3.8) | 1.1 (-2.0 to 4.2) |
|  | **p-value** |  | 0.27 | 0.51 |  |  | 0.71 | 0.47 |
|  | **Cohen's d** |  | 0.20 | 0.11 |  |  | 0.06 | 0.12 |
|  |  |  |  |  |  |  |  |  |
|  | | | | | | | | |
| MIDAS | **Estimate (95% CI)** | 25.6 (8.1 to 43.2) | 17.8 (2.7 to 32.9) | 25.6 (10.4 to 40.7) |  | 39.0 (27.0 to 51.1) | 27.8 (14.6 to 40.9) | 21.5 (8.5 to 34.6) |
|  |  |  | *Versus Control* | |  |  | *Versus Control* | |
|  | **Difference (95% CI)** |  | -7.9 (-30.0 to 14.2) | -0.1 (-22.9 to 22.8) |  |  | -11.3 (-28.1 to 5.6) | -17.5 (-34.7 to -0.3) |
|  | **p-value** |  | 0.48 | 0.99 |  |  | 0.19 | 0.05 |
|  | **Cohen's d** |  | -0.12 | -0.00 |  |  | -0.22 | -0.35 |
| **Diary Measures ᶜ** |  |  |  |  |  |  |  |  |
| Overall health (1-4) | **Estimate (95% CI)** | 2.8 (2.6 to 2.9) | 3.0 (2.9 to 3.1) | 3.1 (3.0 to 3.1) |  | 2.7 (2.6 to 2.7) | 2.9 (2.8 to 3.0) | 2.8 (2.8 to 2.9) |
|  |  |  | *Versus Control* | |  |  | *Versus Control* | |
|  | **Difference (95% CI)** |  | 0.3 (0.1 to 0.4) | 0.3 (0.1 to 0.4) |  |  | 0.3 (0.2 to 0.3) | 0.2 (0.1 to 0.3) |
|  | **p-value** |  | 0.001 | <0.001 |  |  | <0.001 | <0.001 |
|  | **Cohen's d** |  | 0.60 | 0.71 |  |  | 1.02 | 0.64 |
|  |  |  |  |  |  |  |  |  |
| Sleep quality (1-4) | **Estimate (95% CI)** | 2.7 (2.5 to 2.8) | 2.8 (2.7 to 2.9) | 2.7 (2.6 to 2.9) |  | 2.4 (2.4 to 2.5) | 2.6 (2.6 to 2.7) | 2.6 (2.5 to 2.7) |
|  |  |  | *Versus Control* | |  |  | *Versus Control* | |
|  | **Difference (95% CI)** |  | 0.1 (-0.1 to 0.3) | 0.1 (-0.1 to 0.2) |  |  | 0.2 (0.1 to 0.3) | 0.2 (0.1 to 0.3) |
|  | **p-value** |  | 0.26 | 0.51 |  |  | <0.001 | 0.001 |
|  | **Cohen's d** |  | 0.21 | 0.11 |  |  | 0.65 | 0.59 |
|  |  |  |  |  |  |  |  |  |
| Stress (0-10) | **Estimate (95% CI)** | 3.7 (3.3 to 4.1) | 3.0 (2.7 to 3.4) | 2.4 (2.1 to 2.7) |  | 4.0 (3.8 to 4.3) | 3.1 (2.8 to 3.3) | 2.5 (2.2 to 2.8) |
|  |  |  | *Versus Control* | |  |  | *Versus Control* | |
|  | **Difference (95% CI)** |  | -0.6 (-1.1 to -0.1) | -1.3 (-1.7 to -0.8) |  |  | -0.9 (-1.3 to -0.6) | -1.5 (-1.9 to -1.2) |
|  | **p-value** |  | 0.02 | <0.001 |  |  | <0.001 | <0.001 |
|  | **Cohen's d** |  | -0.42 | -0.89 |  |  | -0.93 | -1.51 |
|  |  |  |  |  |  |  |  |  |
| **Whole-Body Pain Scale** | | | | | | | | |
| Number of painful sites ᵈ | **Estimate (95% CI)** | 1.7 (1.4 to 2.1) | 1.4 (1.1 to 1.7) | 1.7 (1.4 to 2.0) |  | 1.7 (1.5 to 1.9) | 1.7 (1.5 to 1.9) | 1.7 (1.5 to 1.9) |
|  |  |  | *Versus Control* | |  |  | *Versus Control* | |
|  | **Difference (95% CI)** |  | -0.3 (-0.8 to 0.2) | -0.01 (-0.4 to 0.4) |  |  | -0.04 (-0.3 to 0.2) | -0.1 (-0.3 to 0.2) |
|  | **p-value** |  | 0.19 | 0.95 |  |  | 0.73 | 0.67 |
|  | **Cohen's d** |  | -0.23 | -0.01 |  |  | -0.05 | -0.07 |
|  |  |  |  |  |  |  |  |  |
| Percent of time with pain ᵉ | **Estimate (95% CI)** | 4.2 (2.6 to 5.7) | 1.9 (0.6 to 3.1) | 4.2 (3.1 to 5.4) |  | 4.7 (3.9 to 5.5) | 3.4 (2.6 to 4.2) | 3.9 (3.1 to 4.7) |
|  |  |  | *Versus Control* | |  |  | *Versus Control* | |
|  | **Difference (95% CI)** |  | -2.3 (-4.2 to -0.4) | 0.1 (-1.8 to 2.0) |  |  | -1.3 (-2.3 to -0.3) | -0.8 (-1.8 to 0.2) |
|  | **p-value** |  | 0.02 | 0.92 |  |  | 0.01 | 0.13 |
|  | **Cohen's d** |  | -0.41 | 0.02 |  |  | -0.40 | -0.25 |
|  |  |  |  |  |  |  |  |  |
| Perceived benefit (overall) ᶠ | **Estimate (95% CI)** | 2.5 (2.1 to 2.9) | 2.3 (1.9 to 2.6) | 2.6 (2.3 to 2.9) |  | 2.7 (2.5 to 2.9) | 2.5 (2.3 to 2.7) | 2.5 (2.3 to 2.7) |
|  |  |  | *Versus Control* | |  |  | *Versus Control* | |
|  | **Difference (95% CI)** |  | -0.2 (-0.7 to 0.3) | 0.1 (-0.4 to 0.6) |  |  | -0.2 (-0.5 to 0.1) | -0.2 (-0.5 to 0.1) |
|  | **p-value** |  | 0.41 | 0.78 |  |  | 0.13 | 0.20 |
|  | **Cohen's d** |  | -0.15 | 0.05 |  |  | -0.27 | -0.24 |
|  |  |  |  |  |  |  |  |  |
| Perceived benefit (headache) ᶠ | **Estimate (95% CI)** | 2.5 (2.0 to 2.9) | 2.1 (1.7 to 2.5) | 2.1 (1.8 to 2.5) |  | 2.5 (2.3 to 2.8) | 2.2 (2.0 to 2.5) | 2.2 (1.9 to 2.4) |
|  |  |  | *Versus Control* | |  |  | *Versus Control* | |
|  | **Difference (95% CI)** |  | -0.4 (-0.9 to 0.2) | -0.3 (-0.9 to 0.2) |  |  | -0.3 (-0.6 to 0.1) | -0.4 (-0.7 to -0.01) |
|  | **p-value** |  | 0.21 | 0.23 |  |  | 0.11 | 0.04 |
|  | **Cohen's d** |  | -0.22 | -0.22 |  |  | -0.29 | -0.37 |
|  |  |  |  |  |  |  |  |  |
| Perceived satisfaction with care ᶠ | **Estimate (95% CI)** | 3.6 (3.1 to 4.1) | 3.9 (3.5 to 4.3) | 3.8 (3.4 to 4.1) |  | 3.6 (3.4 to 3.9) | 3.7 (3.4 to 3.9) | 3.6 (3.3 to 3.9) |
|  |  |  | *Versus Control* | |  |  | *Versus Control* | |
|  | **Difference (95% CI)** |  | 0.3 (-0.3 to 1.0) | 0.2 (-0.4 to 0.8) |  |  | 0.03 (-0.4 to 0.4) | -0.1 (-0.5 to 0.3) |
|  | **p-value** |  | 0.32 | 0.55 |  |  | 0.88 | 0.72 |
|  | **Cohen's d** |  | 0.19 | 0.11 |  |  | 0.03 | -0.06 |

ᵃ Chronic migraine defined as occurring on 15 or more days/month for more than 3 months, which, on at least 8 days/month, has the features of migraine headache. All estimates based on regression models adjusted for baseline value of the respective outcome and recruitment site. Missing data were imputed using multiple imputation procedures.

ᵇ Pain Intensity is measured on a 0-10 scale. All other PROMIS measures are reported as T-score: population average is 50 with a standard deviation of 10.

ᶜ Based on daily diary: Sleep quality and perceived health were measured on a 1-4 scale with 4 indicating better outcomes. Stress was measured on a 0-10 scale. 10=most stress.

ᵈ Poisson regression was performed for the number of painful sites. Group differences are in ratios.

ᵉ Based on a single Likert scale (0-10) question. Each value represents 10% pain, with 10=100% pain.

ᶠ Each question is a 1-5 scale with 5=Much Worse for perceived benefit (overall and headache) and 5=Very Satisfied for satisfaction with care.

**Table S2. Subgroup (heterogeneity) analysis by baseline aura/no aura ᵃ**

|  |  | **No Aura (n=131)** | | |  | **Aura (n=51)** | | |
| --- | --- | --- | --- | --- | --- | --- | --- | --- |
|  |  | **Control (n=42)** | **H3 (n=47)** | **H3-L6 (n=42)** |  | **Control (n=18)** | **H3 (n=14)** | **H3-L6 (n=19)** |
| **PROMIS-29 ᵇ** | | | | | | | | |
| Pain intensity | **Estimate (95% CI)** | 5.1 (4.5 to 5.7) | 4.7 (4.0 to 5.3) | 4.6 (4.0 to 5.2) |  | 5.1 (4.2 to 6.0) | 3.5 (2.4 to 4.5) | 4.4 (3.5 to 5.3) |
|  |  |  | *Versus Control* | |  |  | *Versus Control* | |
|  | **Difference (95% CI)** |  | -0.4 (-1.3 to 0.5) | -0.5 (-1.2 to 0.3) |  |  | -1.6 (-3.0 to -0.3) | -0.7 (-2.0 to 0.6) |
|  | **p-value** |  | 0.38 | 0.24 |  |  | 0.02 | 0.29 |
|  | **Cohen's d** |  | -0.16 | -0.20 |  |  | -0.43 | -0.19 |
|  |  |  |  |  |  |  |  |  |
| Pain interference | **Estimate (95% CI)** | 56.0 (53.8 to 58.2) | 53.9 (51.5 to 56.3) | 54.5 (52.2 to 56.7) |  | 57.0 (53.6 to 60.3) | 49.6 (45.9 to 53.3) | 55.0 (51.8 to 58.2) |
|  |  |  | *Versus Control* | |  |  | *Versus Control* | |
|  | **Difference (95% CI)** |  | -2.1 (-5.3 to 1.1) | -1.5 (-4.6 to 1.6) |  |  | -7.4 (-12.4 to -2.4) | -2.0 (-6.5 to 2.6) |
|  | **p-value** |  | 0.20 | 0.34 |  |  | 0.004 | 0.40 |
|  | **Cohen's d** |  | -0.23 | -0.17 |  |  | -0.53 | -0.15 |
|  |  |  |  |  |  |  |  |  |
| Fatigue | **Estimate (95% CI)** | 53.7 (50.8 to 56.7) | 52.0 (49.1 to 54.9) | 50.3 (47.0 to 53.6) |  | 55.4 (50.8 to 60.0) | 52.6 (48.3 to 56.9) | 53.9 (49.9 to 57.9) |
|  |  |  | *Versus Control* | |  |  | *Versus Control* | |
|  | **Difference (95% CI)** |  | -1.8 (-5.7 to 2.2) | -3.4 (-7.6 to 0.7) |  |  | -2.8 (-8.8 to 3.2) | -1.5 (-7.3 to 4.3) |
|  | **p-value** |  | 0.38 | 0.10 |  |  | 0.36 | 0.61 |
|  | **Cohen's d** |  | -0.15 | -0.28 |  |  | -0.16 | -0.09 |
|  |  |  |  |  |  |  |  |  |
| Anxiety/fear | **Estimate (95% CI)** | 50.0 (47.3 to 52.7) | 49.9 (47.3 to 52.5) | 46.4 (43.8 to 49.0) |  | 52.2 (47.9 to 56.5) | 49.5 (45.2 to 53.8) | 48.7 (44.8 to 52.5) |
|  |  |  | *Versus Control* | |  |  | *Versus Control* | |
|  | **Difference (95% CI)** |  | -0.1 (-3.7 to 3.5) | -3.6 (-7.2 to -0.1) |  |  | -2.7 (-8.5 to 3.0) | -3.6 (-9.3 to 2.2) |
|  | **p-value** |  | 0.95 | 0.04 |  |  | 0.35 | 0.22 |
|  | **Cohen's d** |  | -0.01 | -0.35 |  |  | -0.16 | -0.22 |
|  |  |  |  |  |  |  |  |  |
| Depression/sadness | **Estimate (95% CI)** | 46.0 (43.8 to 48.2) | 46.9 (44.8 to 49.0) | 45.4 (43.2 to 47.6) |  | 45.9 (42.6 to 49.3) | 47.2 (43.7 to 50.7) | 46.4 (43.2 to 49.6) |
|  |  |  | *Versus Control* | |  |  | *Versus Control* | |
|  | **Difference (95% CI)** |  | 0.9 (-1.9 to 3.7) | -0.6 (-3.6 to 2.3) |  |  | 1.3 (-3.3 to 5.9) | 0.4 (-3.9 to 4.8) |
|  | **p-value** |  | 0.53 | 0.67 |  |  | 0.59 | 0.85 |
|  | **Cohen's d** |  | 0.11 | -0.07 |  |  | 0.09 | 0.03 |
|  |  |  |  |  |  |  |  |  |
| Sleep disturbance | **Estimate (95% CI)** | 50.5 (48.9 to 52.2) | 51.2 (49.4 to 52.9) | 50.1 (48.5 to 51.7) |  | 50.3 (47.8 to 52.9) | 49.1 (46.5 to 51.8) | 51.6 (49.2 to 54.1) |
|  |  |  | *Versus Control* | |  |  | *Versus Control* | |
|  | **Difference (95% CI)** |  | 0.6 (-1.7 to 3.0) | -0.5 (-2.7 to 1.8) |  |  | -1.2 (-4.7 to 2.2) | 1.3 (-2.2 to 4.7) |
|  | **p-value** |  | 0.60 | 0.69 |  |  | 0.49 | 0.46 |
|  | **Cohen's d** |  | 0.09 | -0.07 |  |  | -0.12 | 0.13 |
|  |  |  |  |  |  |  |  |  |
| Social roles/activities | **Estimate (95% CI)** | 48.5 (46.2 to 50.8) | 49.1 (46.7 to 51.5) | 49.1 (46.4 to 51.7) |  | 45.7 (42.0 to 49.3) | 51.7 (48.0 to 55.4) | 45.9 (42.3 to 49.6) |
|  |  |  | *Versus Control* | |  |  | *Versus Control* | |
|  | **Difference (95% CI)** |  | 0.6 (-2.5 to 3.7) | 0.5 (-2.9 to 4.0) |  |  | 6.0 (1.0 to 11.1) | 0.3 (-4.6 to 5.1) |
|  | **p-value** |  | 0.72 | 0.76 |  |  | 0.02 | 0.92 |
|  | **Cohen's d** |  | 0.06 | 0.06 |  |  | 0.42 | 0.02 |
|  |  |  |  |  |  |  |  |  |
| Physical function | **Estimate (95% CI)** | 47.6 (45.3 to 50.0) | 47.9 (45.3 to 50.4) | 48.2 (45.7 to 50.7) |  | 45.4 (41.4 to 49.5) | 50.2 (46.3 to 54.1) | 48.3 (44.7 to 51.8) |
|  |  |  | *Versus Control* | |  |  | *Versus Control* | |
|  | **Difference (95% CI)** |  | 0.2 (-3.0 to 3.4) | 0.6 (-2.7 to 3.8) |  |  | 4.8 (-0.5 to 10.1) | 2.8 (-2.5 to 8.1) |
|  | **p-value** |  | 0.89 | 0.73 |  |  | 0.08 | 0.30 |
|  | **Cohen's d** |  | 0.02 | 0.06 |  |  | 0.31 | 0.19 |
|  |  |  |  |  |  |  |  |  |
|  | | | | | | | | |
| MIDAS | **Estimate (95% CI)** | 34.4 (22.3 to 46.4) | 24.6 (12.5 to 36.6) | 24.4 (12.6 to 36.3) |  | 36.9 (16.7 to 57.1) | 23.0 (3.3 to 42.6) | 21.3 (2.2 to 40.4) |
|  |  |  | *Versus Control* | |  |  | *Versus Control* | |
|  | **Difference (95% CI)** |  | -9.8 (-26.3 to 6.7) | -10.0 (-26.0 to 6.1) |  |  | -13.9 (-40.7 to 12.8) | -15.6 (-41.8 to 10.6) |
|  | **p-value** |  | 0.24 | 0.22 |  |  | 0.31 | 0.24 |
|  | **Cohen's d** |  | -0.21 | -0.21 |  |  | -0.18 | -0.20 |
| **Diary Measures ᶜ** |  |  |  |  |  |  |  |  |
| Overall health (1-4) | **Estimate (95% CI)** | 2.7 (2.7 to 2.8) | 2.9 (2.8 to 3.0) | 3.0 (2.9 to 3.0) |  | 2.5 (2.4 to 2.7) | 3.0 (2.9 to 3.2) | 2.8 (2.7 to 2.9) |
|  |  |  | *Versus Control* | |  |  | *Versus Control* | |
|  | **Difference (95% CI)** |  | 0.2 (0.1 to 0.3) | 0.2 (0.1 to 0.3) |  |  | 0.5 (0.4 to 0.6) | 0.2 (0.1 to 0.4) |
|  | **p-value** |  | <0.001 | <0.001 |  |  | <0.001 | <0.001 |
|  | **Cohen's d** |  | 0.64 | 0.89 |  |  | 1.18 | 0.61 |
|  |  |  |  |  |  |  |  |  |
| Sleep quality (1-4) | **Estimate (95% CI)** | 2.6 (2.5 to 2.7) | 2.6 (2.6 to 2.7) | 2.7 (2.6 to 2.7) |  | 2.3 (2.2 to 2.4) | 2.8 (2.7 to 2.9) | 2.7 (2.6 to 2.8) |
|  |  |  | *Versus Control* | |  |  | *Versus Control* | |
|  | **Difference (95% CI)** |  | 0.1 (-0.1 to 0.2) | 0.1 (-0.03 to 0.2) |  |  | 0.5 (0.3 to 0.7) | 0.4 (0.2 to 0.5) |
|  | **p-value** |  | 0.36 | 0.19 |  |  | <0.001 | <0.001 |
|  | **Cohen's d** |  | 0.17 | 0.23 |  |  | 0.99 | 0.76 |
|  |  |  |  |  |  |  |  |  |
| Stress (0-10) | **Estimate (95% CI)** | 3.6 (3.3 to 3.8) | 3.2 (3.0 to 3.5) | 2.5 (2.2 to 2.7) |  | 4.7 (4.4 to 5.1) | 2.6 (2.2 to 3.0) | 2.4 (2.1 to 2.8) |
|  |  |  | *Versus Control* | |  |  | *Versus Control* | |
|  | **Difference (95% CI)** |  | -0.3 (-0.7 to -0.01) | -1.1 (-1.4 to -0.8) |  |  | -2.1 (-2.7 to -1.6) | -2.3 (-2.8 to -1.8) |
|  | **p-value** |  | 0.05 | <0.001 |  |  | <0.001 | <0.001 |
|  | **Cohen's d** |  | -0.35 | -1.13 |  |  | -1.38 | -1.57 |
|  |  |  |  |  |  |  |  |  |
| **Whole-Body Pain Scale** | | | | | | | | |
| Number of painful sites ᵈ | **Estimate (95% CI)** | 1.7 (1.5 to 1.9) | 1.6 (1.4 to 1.8) | 1.7 (1.5 to 1.9) |  | 1.8 (1.6 to 2.1) | 1.6 (1.3 to 1.9) | 1.8 (1.5 to 2.1) |
|  |  |  | *Versus Control* | |  |  | *Versus Control* | |
|  | **Difference (95% CI)** |  | -0.1 (-0.4 to 0.2) | -0.03 (-0.3 to 0.2) |  |  | -0.2 (-0.6 to 0.2) | -0.1 (-0.4 to 0.3) |
|  | **p-value** |  | 0.57 | 0.83 |  |  | 0.28 | 0.77 |
|  | **Cohen's d** |  | -0.09 | -0.03 |  |  | -0.18 | -0.05 |
|  |  |  |  |  |  |  |  |  |
| Percent of time with pain ᵉ | **Estimate (95% CI)** | 4.5 (3.7 to 5.4) | 3.0 (2.2 to 3.8) | 4.4 (3.6 to 5.2) |  | 4.4 (3.1 to 5.8) | 2.8 (1.5 to 4.2) | 3.3 (2.1 to 4.5) |
|  |  |  | *Versus Control* | |  |  | *Versus Control* | |
|  | **Difference (95% CI)** |  | -1.5 (-2.6 to -0.5) | -0.1 (-1.2 to 1.0) |  |  | -1.6 (-3.4 to 0.2) | -1.1 (-2.8 to 0.7) |
|  | **p-value** |  | 0.006 | 0.83 |  |  | 0.08 | 0.22 |
|  | **Cohen's d** |  | -0.47 | -0.04 |  |  | -0.30 | -0.21 |
|  |  |  |  |  |  |  |  |  |
| Perceived benefit (overall) ᶠ | **Estimate (95% CI)** | 2.7 (2.5 to 2.9) | 2.4 (2.2 to 2.7) | 2.5 (2.3 to 2.7) |  | 2.6 (2.2 to 3.0) | 2.4 (2.0 to 2.8) | 2.7 (2.4 to 3.0) |
|  |  |  | *Versus Control* | |  |  | *Versus Control* | |
|  | **Difference (95% CI)** |  | -0.2 (-0.5 to 0.1) | -0.2 (-0.5 to 0.1) |  |  | -0.2 (-0.7 to 0.3) | 0.1 (-0.4 to 0.6) |
|  | **p-value** |  | 0.13 | 0.18 |  |  | 0.50 | 0.69 |
|  | **Cohen's d** |  | -0.27 | -0.25 |  |  | -0.12 | 0.07 |
|  |  |  |  |  |  |  |  |  |
| Perceived benefit (headache) ᶠ | **Estimate (95% CI)** | 2.6 (2.3 to 2.8) | 2.2 (2.0 to 2.5) | 2.1 (1.8 to 2.3) |  | 2.4 (2.0 to 2.8) | 2.2 (1.7 to 2.6) | 2.3 (1.9 to 2.7) |
|  |  |  | *Versus Control* | |  |  | *Versus Control* | |
|  | **Difference (95% CI)** |  | -0.3 (-0.7 to 0.02) | -0.5 (-0.9 to -0.1) |  |  | -0.3 (-0.8 to 0.3) | -0.1 (-0.6 to 0.5) |
|  | **p-value** |  | 0.06 | 0.007 |  |  | 0.38 | 0.78 |
|  | **Cohen's d** |  | -0.34 | -0.50 |  |  | -0.16 | -0.05 |
|  |  |  |  |  |  |  |  |  |
| Perceived satisfaction with care ᶠ | **Estimate (95% CI)** | 3.6 (3.3 to 3.9) | 3.8 (3.5 to 4.0) | 3.7 (3.4 to 4.0) |  | 3.7 (3.2 to 4.1) | 3.6 (3.1 to 4.0) | 3.4 (3.0 to 3.9) |
|  |  |  | *Versus Control* | |  |  | *Versus Control* | |
|  | **Difference (95% CI)** |  | 0.2 (-0.2 to 0.6) | 0.1 (-0.3 to 0.5) |  |  | -0.1 (-0.8 to 0.5) | -0.2 (-0.9 to 0.4) |
|  | **p-value** |  | 0.33 | 0.52 |  |  | 0.70 | 0.42 |
|  | **Cohen's d** |  | 0.18 | 0.12 |  |  | -0.07 | -0.14 |

^a^ Participants were classified as having migraine with aura if they reported aura with any migraine attacks. Typically, participants reported an aura with most of their migraine attacks.

ᵇ Pain Intensity is measured on a 0-10 scale. All other PROMIS measures are reported as T-scores: population average is 50 with a standard deviation of 10.

ᶜ Based on daily diary: Sleep quality and perceived health were measured on a 1-4 scale with 4 indicating better outcomes. Stress was measured on a 0-10 scale. 10=most stress.

ᵈ Poisson regression was performed for the number of painful sites. Group differences are in ratios.

ᵉ Based on a single Likert scale (0-10) question. Each value represents 10% pain, with 10=100% pain.

ᶠ Each question is a 1-5 scale with 5=Much Worse for perceived benefit (overall and headache) and 5=Very Satisfied for satisfaction with care.
